# Redefining the practical roles of psychiatrists in epilepsy care: A framework for collaboration in Japan

**DOI:** 10.1002/pcn5.70188

**Published:** 2025-08-14

**Authors:** Go Taniguchi, Hirotaka Iwaki, Izumi Kuramochi, Toru Horinouchi, Shunsuke Takagi

**Affiliations:** ^1^ Department of Epileptology National Center of Neurology and Psychiatry Tokyo Japan; ^2^ Department of Psychiatry Douousato Hospital Hokkaido Japan; ^3^ Department of Psychiatry Hokkaido University Hokkaido Japan; ^4^ Department of Psychiatry Institute of Science Tokyo Tokyo Japan

**Keywords:** antiseizure medications, epilepsy, multidisciplinary collaboration, psychiatric comorbidities, psychosocial support

## Abstract

Psychiatric symptoms are prevalent among people with epilepsy (PWE), yet psychiatric care remains underdeveloped in epilepsy services worldwide. Many psychiatrists lack sufficient familiarity with epilepsy, contributing to gaps in care. Japan, however, has a distinctive history where psychiatrists played a central role in epilepsy treatment, especially in managing epilepsy‐related psychosis. This legacy, though fading, offers valuable insights. This review proposes a renewed framework to reestablish the psychiatrist's role in epilepsy care, informed by Japan's historical context and current global trends. The framework consists of five domains: (1) the historical relationship between psychiatry and epilepsy; (2) diagnosis and treatment of psychiatric symptoms in PWE; (3) psychosocial interventions; (4) interdisciplinary collaboration; and (5) future directions in training, research, policy, and clinical integration. While psychiatry's role in epilepsy has diminished in many countries, Japan may still retain structural and cultural foundations for reintegration. By redefining psychiatric involvement, we aim to inspire general psychiatrists and trainees to engage with epilepsy care. Reaffirming the psychiatric perspective is essential for delivering comprehensive, patient‐centered care to PWE.

## INTRODUCTION

### Historical background of epilepsy care in Japan

Japan has a unique epilepsy treatment system compared to other countries.

Kure (1865–1932), a psychiatrist who studied under Emil Kraepelin (1856–1926), and Miura (1864–1950), a neurologist who studied under Jean‐Martin Charcot (1825–1893), jointly founded the former Japan Society of Neurology (JSN) in 1902.^1^ Both psychiatry and neurology were involved in the treatment of epilepsy.^1^ In 1935, the Japan Society of Neurology changed its name to the Japanese Society of Psychiatry and Neurology (JSPN).^1^, ^2^ Gradually, the number of neurologists in the society declined, and it became composed almost exclusively of psychiatrists. This shift likely occurred because, at that time, there were no neurology departments or courses in Japan, whereas departments of psychiatry had already been established at various universities.^2^ Consequently, the treatment and research of neurological disorders were dispersed among internal medicine, psychiatry, and orthopedics.^2^ In 1960, neurology separated from the JSPN to form its own society, now the JSN.^2^ However, psychiatry continued to play a central role in the treatment and research of epilepsy for some time thereafter.^1^

### Rise and decline of psychiatry's role

When the Japan Epilepsy Study Group was established in 1967, its founding members included several psychiatrists, including its first president, Haruo Akimoto (1906–2007).^3^ In 1975, psychiatrists Toyoji Wada (1919–2002) and Masakazu Seino (1930–2007) played pivotal roles in establishing Japan's first dedicated epilepsy center.^3^ This center delivered comprehensive epilepsy care at a time when the concept of comprehensive medicine had not yet been formally recognized in Japan.

**Figure 1:**
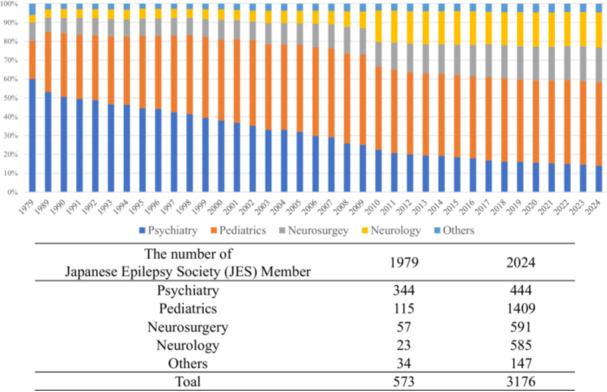
Changes in the medical specialty composition of the Japanese Epilepsy Society (JES) membership between 1979 and 2024. Trends in the percentage of each department from the establishment of the Japan Epilepsy Society (1979) to the present (2024). When the Japanese Epilepsy Society was founded in 1979, psychiatrists accounted for 60% of its members, but the percentage has gradually declined to 14% as of 2024.

The Japan Epilepsy Study Group evolved into the Japanese Epilepsy Society (JES) in 1979.^3^ At the time of its establishment, membership included 344 in psychiatry, 115 in pediatrics, 57 in neurosurgery, 23 in neurology, and 34 in other fields (Figure 1).^3^ Subsequently, various administrative patient support systems were established based on the premise that psychiatrists would examine patients, and epilepsy remains classified as a psychiatric disorder under medical policy framework.

While psychiatrists historically played a central role in managing seizures and psychiatric symptoms in adults with epilepsy, their involvement has steadily declined. JES membership among psychiatrists peaked at 656 in 1992 but had fallen to 444 by 2024. In contrast, memberships from other specialties have steadily increased, reaching 1409 in pediatrics, 585 in neurosurgery, and 591 in neurology^4^ (Figure 1). Today, psychiatry represents the smallest clinical discipline within the JES.

In addition, there are virtually no university psychiatry departments in Japan with epilepsy as a major research focus. According to a survey conducted in 2024, only a minority of psychiatrists (18.9%)^1^ had seen a new patient with epilepsy in the past three months, and the majority (89.2%) reported feeling uncomfortable with epilepsy treatment.^5^

### Global trends and Japan lagging

Globally, the modern history of epilepsy treatment began when neurologist John Hughlings Jackson (1835–1911) established the mechanism and concept of epilepsy as a neurological disease in 1870.^3^ Since then, many countries have developed healthcare systems centered around neurologists. Epilepsy has long been defined as a chronic neurological disease characterized by recurrent seizures, with psychiatric symptoms treated as secondary conditions by various specialists.^3^ However, since the early 2000s, experts—particularly in Europe and the United States—have shown increasing interest in the quality of life (QOL) of people with epilepsy (PWE), focusing on depression and other psychiatric symptoms, cognitive function, employment and schooling, and stigma.^3^ In 2005, the International League Against Epilepsy (ILAE) redefined epilepsy as: “Epilepsy is a disorder of the brain characterized by an enduring predisposition to generate epileptic seizures (ES) and by the neurobiological, cognitive, psychological, and social consequences of this condition.”^6^

This definition explicitly states that psychosocial problems, including psychiatric symptoms—once considered secondary comorbidities—are intrinsic to epilepsy itself. In 2017, the Neuropsychiatric Commission of the ILAE emphasized the need for comprehensive training of epileptologists regarding psychiatric symptoms.^7^ Today, specialists around the world continue to address psychosocial issues in epilepsy, but concern remains that Japan may be lagging as many psychiatrists retire from active involvement.^3^ Although Japan has published many excellent studies on epilepsy‐related psychosis, it has produced few international studies on depression and psychogenic non‐epileptic seizures (PNES), which are currently of global interest.^3^

### Psychiatry in epilepsy: Global gaps and Japan's collaborative foundations

Despite international consensus on the importance of addressing psychiatric comorbidities in epilepsy, real‐world implementation remains inconsistent across countries. In most settings, psychiatric support is fragmented or absent, and comorbidities such as depression, anxiety, psychosis, and PNES often go unrecognized or undertreated.^7^, ^8^, ^9^, ^10^ Notably, however, countries such as Italy have adopted a more integrated approach: neuropsychiatrists—clinicians trained in both neurology and psychiatry—manage epilepsy care across childhood, adolescence, and early adulthood.^11^ This “lumper” model facilitates early detection and continuous management of psychiatric and psychological comorbidities, helping to overcome the fragmentation typically seen in adult services. Such frameworks may serve as valuable references for developing comprehensive, life‐course models of epilepsy care.

By contrast, many countries have adopted a “splitter” model, in which neurologists and psychiatrists operate in silos, particularly in adult care. Recent reports from France and the United States have described similar challenges, particularly regarding the lack of structured collaboration between neurologists and psychiatrists.^8^, ^9^ Conditions such as PNES—often misdiagnosed and stigmatized—are emblematic of the difficulties posed by the current separation of disciplines. Without clear clinical pathways for shared care, patients are often caught in a diagnostic and therapeutic limbo between neurology and psychiatry.^7^, ^12^ Moreover, Murphy et al. argue that fundamental epistemological divides persist, as neurology and psychiatry rarely overlap in training, research, or clinical culture.^12^

In response to these systemic limitations, the literature has increasingly called for neurologists, especially epileptologists, to take on greater responsibility in the detection and management of psychiatric symptoms.^8^, ^9^, ^10^ Practical guidelines have been developed to support neurologist‐led screening and pharmacologic treatment for common psychiatric disorders, including depression, anxiety, and peri‐ictal psychosis.^10^ While these efforts reflect a pragmatic response to workforce shortages and access barriers, they also underscore the pressing need for integrated, interdisciplinary care models rather than reliance on stopgap measures.^9^, ^10^

Nevertheless, when considering the current landscape of epilepsy care in Japan, a more nuanced picture emerges. While the number of psychiatrists actively affiliated with epilepsy societies has indeed declined, a distinct subset of psychiatrists who specialize in epilepsy remains—rooted in Japan's unique historical context of psychiatry‐epilepsy integration. As previously discussed, Japan has produced a rich body of clinical work on epilepsy‐related psychosis, and this legacy continues to influence psychiatric practice. Furthermore, since 2015, Japan has implemented a national Epilepsy Support Center Hospital initiative under the Ministry of Health, Labour and Welfare. This program mandates the participation of both certified epileptologists and psychiatrists in its multidisciplinary framework, thereby institutionalizing collaboration. Although Japan faces similar structural challenges as other countries, such frameworks suggest that the foundation for psychiatrist–epileptologist collaboration may be more robust than commonly assumed. If further developed and evaluated, Japan's integrated approach—anchored in both historical precedent and national policy—could serve as a valuable model for countries seeking to bridge the longstanding divide between neurology and psychiatry in epilepsy care.

### Reaffirming the role of psychiatry in epilepsy care

Given Japan's historical context and current global trends, it is both timely and necessary to re‐examine the role of psychiatry in the comprehensive management of epilepsy. This review aims to: (1) clarify the roles psychiatrists are expected to fulfill in epilepsy care; (2) reduce the perceived barriers to psychiatric involvement, particularly among general psychiatrists and trainees; and (3) contribute to global efforts to reintegrate psychiatric perspectives into epilepsy services, thereby addressing the widespread marginalization of psychiatry observed in many countries.

A recently published Japanese guidebook on epilepsy care delineated the roles of psychiatrists into four main categories: (1) the treatment of epileptic seizures (ES); (2) the differential diagnosis between epilepsy and psychiatric disorders; (3) the diagnosis and treatment of psychiatric comorbidities in epilepsy; and (4) the provision of psychosocial support for individuals with epilepsy.^13^ Importantly, the required expertise, target populations, and clinical approaches differ markedly between the first two and the latter two categories. While the management of seizures and diagnostic clarification often requires specialized training in epileptology, the third and fourth domains offer a broader scope for psychiatrists—including those without epilepsy‐specific expertise—to make meaningful contributions.

This review will primarily focus on the latter two domains. Chapter 2 examines the diagnosis and treatment of psychiatric symptoms comorbid with epilepsy, such as depression, anxiety, and psychosis. Chapter 3 discusses psychosocial challenges, including stigma, social isolation, and barriers to employment or education, and presents strategies for psychiatric and social support. Chapter 4 explores interdisciplinary collaboration between psychiatrists and other healthcare professionals, and Chapter 5 outlines future directions for clinical care, training, and research. Through this structure, we aim to reaffirm the critical role that psychiatry can and should play in the evolving field of epilepsy care.

## DIAGNOSIS AND TREATMENT OF PSYCHIATRIC SYMPTOMS IN PWE

### Co‐existing psychiatric symptoms

Psychiatric disorders frequently coexist with epilepsy. A recent meta‐analysis found that the prevalence of psychiatric comorbidities among PWE is as high as 43.3%.^14^ Compared to healthy individuals, the incidence rate is two to five times higher. Furthermore, the relationship between epilepsy and psychiatric disorders is bidirectional.^15^ Given the strong connection between the two, it is essential for general psychiatrists to understand the specific psychiatric features and care needs associated with epilepsy.

#### Depression

Depression is the most common psychiatric disorder in PWE, with a lifetime prevalence of 30%–35%.^16^ Several cross‐sectional studies have also found a higher prevalence (21%–33%) in patients with seizures compared with those without seizures (4%–6%).^17^, ^18^, ^19^ Despite the high incidence of depression in PWE, it remains under‐recognized and under‐treated. For example, in a study of 97 PWE with major depressive episodes, more than half took over a year to consult a psychiatrist.^20^ In clinical practice, clinicians often do not adequately inquire about comorbidities.^21^

The bidirectional relationship between epilepsy and depression is well documented. Regarding depression in PWE, a study^22^ using the United Kingdom General Practice Research Database of 3773 PWE and 14,025 control participants found that the incidence of depression in the epilepsy group was significantly higher than in the control group during the 3 years before and after epilepsy onset. Similarly, the incidence of suicide was elevated in the 3 years preceding and in the year following epilepsy diagnosis. Conversely, regarding epilepsy in patients with depression, a Swedish study comparing 1885 patients with a history of hospitalization and 15,080 controls found that the age‐adjusted odds ratio for non‐induced seizures was significantly elevated: 2.5 for major depressive disorder and 2.6 for suicide attempts.^23^

Recommendations for managing depression, based on expert consensus,^24^ include the use of the Neurological Disorders Depression Inventory for Epilepsy (NDDI‐E) for screening, with a cutoff score of 13. Diagnosis is based on the International Classification of Diseases (ICD)‐10. Suicide risk should be assessed, and adjustments to Anti‐seizure medications (ASMs) should be considered if a causal relationship with depressive episodes is suspected. The risk‐benefit balance should guide the use of antidepressants, with selective serotonin reuptake inhibitors (SSRIs) and serotonin noradrenaline reuptake inhibitors (SNRIs) typically serving as first‐line agents. Treatment should continue for ≥6 months for a first episode and ≥9 months for recurrent or severe depression.

Few studies have assessed the effects of antidepressants on seizure frequency in PWE. However, a Cochrane review found no evidence that antidepressant use significantly increases seizure frequency compared with controls.^25^ In addition to these bidirectional associations, the potential impact of ASMs on suicidality is an important clinical concern and is discussed further in the “Suicide risk and regulatory warnings“ section.

#### Psychosis in Epilepsy–Interictal Psychosis

Interictal psychosis is characterized by the absence of a temporal relationship between psychotic symptoms and seizures. Initially, Slater referred to it as “schizophrenia‐like psychosis in epilepsy,”^26^, ^27^ and later, Toone introduced the term “interictal psychosis” to distinguish it from postictal psychosis (PIP).^28^ Historically, interictal psychosis was considered a distinct condition associated with milder emotional symptoms, minimal personality deterioration, and religious‐themed delusions and hallucinations.^27^, ^29^, ^30^ However, over time, controlled studies using structured assessment methods, such as the Positive and Negative Syndrome Scale and Brief Psychiatric Rating Scale, have not identified clear qualitative differences between interictal psychosis and schizophrenia.^31^, ^32^, ^33^, ^34^, ^35^, ^36^ Although these studies had limitations, including small sample sizes and inadequate control of confounding clinical variables, they are important because they utilized standardized methods, reduced interrater variability, and were conducted by multiple research groups.

Given the absence of clear qualitative differences, differentiating interictal psychosis from schizophrenia remains challenging. Adachi and Akanuma proposed evaluating contributing factors in terms of epilepsy‐related and general congenital vulnerabilities.^37^ Epilepsy‐related factors include age of onset, focal epilepsy, focal impaired awareness seizures, and ASM use. General vulnerabilities include intellectual ability, family history of psychosis, and educational attainment.^38^, ^39^ In a large population‐based study, a family history of schizophrenia was associated with an increased risk of schizophrenia (relative risk [RR] 7.57) and schizophrenia‐like psychosis (RR 4.03) in PWE.^40^ Therefore, a reasonable interpretation is that the predominance of epilepsy‐related factors suggests interictal psychosis, whereas the predominance of congenital vulnerabilities suggests schizophrenia. However, because epilepsy‐related vulnerabilities are common in PWE, this distinction alone may be insufficient to exclude schizophrenia. Currently, there is no clear boundary between these conditions, and they may exist along a continuum.

Pharmacotherapy for interictal psychosis is similar to that for schizophrenia. Antipsychotic selection should balance efficacy and side effects. No significant differences in efficacy among antipsychotics at equivalent chlorpromazine doses have been observed.^41^ Although antipsychotics are known to have seizure‐inducing potential, most do not worsen seizure frequency. In fact, some prospective studies suggest a reduction in seizures in PWE treated with antipsychotics.^42^ No significant differences in seizure exacerbation have been found between first‐ and second‐generation antipsychotics, and none of the six first‐generation or four second‐generation antipsychotics caused more seizures than the others.^42^ Therefore, it is reasonable to consider second‐generation agents, as in schizophrenia treatment, rather than limiting to first‐generation agents. The choice of drug should be guided by the risk‐benefit ratio according to the patient's situation.

#### Psychosis in epilepsy–alternative psychosis

Alternative psychosis refers to a psychotic episode occurring when a patient with intractable chronic epilepsy suddenly experiences seizure freedom.^43^ Heinrich Landolt first referred to the disappearance of epileptiform activity on electroencephalography (EEG) in patients with drug‐resistant epilepsy accompanied by acute behavioral deterioration in 1953 and called it forced normalization (FN). Subsequently, Hans Tellenbach shifted the concept to a clinical perspective, calling it “alternative psychosis,” and proposed that epilepsy in patients with FN does not actually disappear. In 1991, Peter Wolf coined the term “paradoxical normalization,” referring to cases where EEG and epileptic activity returned to normal but clinical deterioration persisted. He suggested that epilepsy remained active in the subcortex and that epileptic discharges could induce psychosis through alternative pathways.^44^

The emergence of alternative psychosis is triggered by suppression of ES. Various treatments, including ASMs, focal resection, and vagus nerve stimulation, have been reported to induce alternative psychosis.^43^

In terms of treatment, medications that trigger the emergence of alternative psychosis are often reduced or discontinued, or antipsychotic medications are introduced.

In one systematic review,^44^ the remission rate of psychiatric symptoms was 56.2% in patients treated with antipsychotics and 92.8% in those not treated with antipsychotics, suggesting that antipsychotic use is not a clear determinant of recovery. Conversely, complete resolution was achieved in 87% of patients who discontinued ASMs and 75% of patients who did not, indicating that reducing or discontinuing ASMs is an important factor in treatment.

### Psychiatric adverse effects of ASMs: Clinical characteristics, detection challenges, and management strategies

ASMs play a central role in epilepsy management; however, they are associated with psychiatric adverse effects that significantly impair QOL^45^ and, in severe cases, may lead to life‐threatening outcomes such as suicidal behavior or aggression. This section reviews current evidence regarding the clinical characteristics, diagnostic challenges, and management strategies related to ASM‐induced psychiatric complications.

#### Clinical spectrum of psychiatric adverse effects

ASMs exhibit psychotropic properties. For example, sodium valproate and carbamazepine demonstrate mood‐stabilizing effects and may suppress manic symptoms, whereas lamotrigine is associated with a lower risk of depressive symptoms and possesses mood‐stabilizing properties.^45^, ^46^ Conversely, many ASMs, including newer agents, can induce mood disorders such as depressive episodes or psychotic symptoms resembling schizophrenia. The mechanisms underlying these psychiatric effects may involve enhanced gamma‐aminobutyric acid neurotransmission, folate deficiency due to polypharmacy, and other factors.^46^

A large retrospective analysis conducted in the United States involving 4,085 adults found that 17% of patients experienced psychiatric symptoms.^47^ Although sodium channel blockers have been suggested to have relatively fewer psychiatric adverse effects,^47^ direct comparative studies providing robust evidence are lacking.

Commonly prescribed ASMs can be broadly categorized into two groups based on their psychiatric adverse effect profiles: (1) levetiracetam and perampanel, associated with irritability, anger, and aggression^47^, ^48^; and (2) topiramate and zonisamide, associated with cognitive dysfunction, depressive symptoms, and psychotic symptoms such as hallucinations and delusions.^47^, ^48^

Among ASMs, those most frequently requiring discontinuation due to psychiatric adverse effects include perampanel, topiramate, zonisamide, and levetiracetam.^49^ Brivaracetam is considered to carry a lower risk of psychiatric adverse effects than levetiracetam; however, it still presents a risk of irritability and aggression, necessitating careful monitoring.^48^, ^49^, ^50^ An overview of the psychiatric adverse effects associated with each ASM is summarized in Table 1.^15^, ^46^

**Table 1:** Common psychiatric adverse effects associated with antiseizure medications (based on references 15 and 46).

Drugs	Psychiatric adverse effects	Risk factors
Barbiturates (phenobarbital/primidone)	Depression (hyperactivity, irritability, and aggression)	Children and individuals with intellectual disabilities
Benzodiazepine	(Hyperactivity, irritability, and aggression)	Children and individuals with intellectual disabilities
Brivaracetam	Aggressive behavior, depression, and psychosis	
Carbamazepine	−	
Eslicarbazepine	−	
Ethosuximide	Psychosis	
Felbamate	Anxiety and psychosis	
Gabapentin	(Hyperactivity, irritability, and aggression)	Children and individuals with intellectual disabilities
Lacosamide	−	
Lamotrigine	(Hyperactivity, irritability, and aggression)	Individuals with intellectual disabilities
Levetiracetam	Irritability, aggression, anxiety, depression, and psychosis	
Oxcarbazepine	−	
Perampanel	Irritability, aggression, anxiety, depression, and psychosis	Blood concentration dependence
Phenytoin	Psychosis	At high serum levels
Pregabalin	Depression	
Rufinamide	−	
Stiripentol	Hyperactivity, irritability, and aggression	
Tiagabine	Irritability	
Topiramate	Depression, psychosis, irritability, and cognitive dysfunction	Dose dependent
Valproic acid	−	
Vigabatrin	(Hyperactivity, aggression, and agitation)	Children and individuals with intellectual disabilities
Zonisamide	Depression, psychosis, irritability, and cognitive dysfunction	Dose dependent

#### Suicide risk and regulatory warnings

The US Food and Drug Administration (FDA) has issued a black‐box warning for all ASMs regarding the increased risk of suicide. However, there is no consensus on which specific ASMs carry the highest risk, partly due to variations in study designs.^15^, ^51^, ^52^, ^53^ Some studies, including those conducted by the FDA, did not specify the underlying conditions of the patients, introducing potential confounding factors. For example, ASMs are frequently prescribed as mood stabilizers in patients with bipolar disorder, suggesting that psychiatric comorbidities may have influenced the outcomes.

Recent studies have highlighted the complex, bidirectional relationship between suicide and epilepsy, emphasizing the multifactorial nature of this risk. In response, the ILAE has noted the limitations of the FDA meta‐analysis underlying the black‐box warnings and emphasized the importance of screening high‐risk individuals and implementing preventive strategies.^54^

#### Risk factors and the possibility of severe outcomes

Severe psychiatric adverse effects can escalate to suicide or violent behavior. One of the primary challenges is the difficulty in early detection of these symptoms. Identifying patients predisposed to psychiatric adverse effects is particularly important. Patients with pre‐existing psychiatric disorders (such as depression and schizophrenia) and intellectual disabilities are at increased risk.^55^, ^56^, ^57^, ^58^ Furthermore, recent studies suggest that female patients may also face an elevated risk.^59^, ^60^

When initiating ASMs, a “start low, go slow” titration strategy is generally recommended. This approach has been shown to reduce the risk of severe idiosyncratic reactions and improve tolerability, particularly regarding common central nervous system‐related adverse effects.^61^ However, psychiatric adverse effects, especially dose‐dependent ones, may still occur even with slow titration. Therefore, clinicians should not assume that gradual dose increases alone will prevent psychiatric complications.^62^

#### Challenges in detection and diagnosis

Early detection of psychiatric adverse effects associated with ASMs is complicated by overlapping clinical features between epilepsy and ASM‐induced symptoms. For instance, postictal confusion and transient cognitive changes can mimic psychiatric side effects.^45^, ^46^ In addition, patients and caregivers often misinterpret gradual mood or behavioral changes as personality shifts rather than drug‐related adverse effects.

Underreporting is further exacerbated by the subtle nature of early symptoms, which may delay appropriate intervention until severe outcomes, such as suicide attempts, occur. Standardized tools such as the Patient Health Questionnaire‐9 and the NDDI‐E for depression, as well as the Neuropsychiatric Inventory for broader symptom assessment, are critical for timely identification of psychiatric adverse effects.^63^, ^64^ Furthermore, caregiver interviews are indispensable, as caregivers are often the first to detect subtle behavioral changes that patients themselves may not recognize.^58^

### Psychiatric symptoms specific to PWE

This section focuses on key psychiatric symptoms specific to epilepsy that psychiatrists should recognize and address in clinical practice.

#### Postictal Psychosis (PIP)

PIP is a rare but severe psychiatric complication that occurs in approximately 2% of PWE.^65^ It typically presents as a delusional episode with hallucinations following a lucid interval after a cluster of focal seizures, with or without secondary generalization into bilateral tonic‐clonic seizures. The most severe consequences include self‐directed or other‐directed aggression, underscoring its medicolegal relevance.

In 1988, Logsdail and Toone proposed diagnostic criteria for PIP^66^:

- Onset of confusion or psychosis within 1 week of the return of apparently normal mental function
- Duration of 1 day to 3 months
- Mental state characterized by:

a) Clouding of consciousness, disorientation, or delirium
b) Delusions or hallucinations in clear consciousness
c) A mixture of (a) and (b)

- No evidence of factors such as:

a) ASM toxicity
b) A previous history of interictal psychosis
c) EEG evidence of status epilepticus
d) Recent head injury or substance intoxication

Patients with PIP often exhibit manic moods, religious or paranoid delusions, agitation, irritability, and impulsivity, resulting in self‐harm or violence. Most cases resolve spontaneously within an average of 9 days, with complete remission typically occurring within a month.

##### Clinical features and diagnostic challenges

PIP typically follows a lucid interval lasting 12–120 h, distinguishing it from postictal delusional confusion. It often remains underdiagnosed, particularly in patients living alone or experiencing nocturnal seizures. Symptoms include thymic disturbances, hallucinations, and delusions, commonly persecutory, religious, or grandiose in nature. Episodes tend to be stereotyped in the same patient and resolve abruptly.

##### Risk factors and pathophysiology

PIP primarily affects patients with refractory focal epilepsy, particularly temporal lobe epilepsy of >10 years duration.^67^ Seizures in these individuals often exhibit secondary bilateral spread. EEG recordings during PIP are usually indistinguishable from interictal EEGs, aiding in the exclusion of ongoing seizure activity. Six primary risk factors have been identified: male sex, family history of psychiatric illness, interictal EEG abnormalities, epilepsy of encephalitic origin, extensive temporal lesions, and right temporal discharges.^67^ Genetic and neuroanatomical vulnerabilities are considered contributory but remain under investigation.

##### Management and prognosis

PIP is considered an autonomous condition, as EEG recordings during episodes typically lack ictal activity, as EEGs recorded during episodes typically lack ictal discharges.61 Recent experimental studies support this distinction, demonstrating that postictal behavioral impairments may result from prolonged hypoperfusion and hypoxia in seizure‐affected brain regions. These changes, mediated by COX‐2‐dependent vasoconstriction, have been shown to impair memory and behavior in animal models^68^, ^69^ Although acute episodes typically resolve spontaneously, antipsychotic medication and inpatient psychiatric care may be necessary in cases of agitation or risk to self or others. Long‐term seizure control, through either medical or surgical interventions, effectively eliminates the risk of recurrence, underscoring the causal role of seizures. Although severe, PIP is treatable with appropriate management.

#### Ictal fear (IF)

ES may manifest as a subjective experience of fear, known as IF. IF involves sudden, unprovoked fear during a seizure, independent of actual or perceived threats.^70^ It is often accompanied by autonomic symptoms such as tachycardia, sweating, and dissociation, closely resembling panic attacks.

##### Neuroanatomy and mechanisms of IF

IF is commonly associated with seizure activity in the amygdala and anterior hippocampus, key structures of the limbic system.^71^ It is typically linked to epileptic discharges in the temporal lobe, particularly the amygdala, hippocampus, and parahippocampal gyrus. The amygdala mediates fear responses, integrating sensory input with autonomic and endocrine output, while the hippocampus contributes contextual memory. Intracranial EEG studies have also identified IF originating from extratemporal sites such as the middle cingulate gyrus, demonstrating the diverse neuroanatomical basis of fear generation.^72^

##### Clinical features and differential diagnosis

IF typically occurs at seizure onset and is marked by autonomic arousal, agitation, or immobilization. It may also involve vivid hallucinations or complex behaviors such as crying out for help. Distinguishing IF from panic attacks is crucial, as panic attacks are spontaneous and often include anticipatory anxiety.

##### Treatment and management

Management of IF centers on achieving seizure control. ASMs targeting temporal lobe activity and surgical interventions in refractory cases often eliminate IF. When differentiation from psychiatric conditions is difficult, prolonged EEG monitoring and functional neuroimaging are essential.

#### Interictal dysphoric disorder (IDD)

IDD refers to a cluster of chronic affective and somatic symptoms in PWE. These include dysphoria, irritability, fear, anxiety, insomnia, anergia, headaches, pain, and occasional euphoria. Dysphoria, marked by irritability and depressive mood, is a hallmark of IDD. Irritability may appear as persistent frustration or sudden outbursts without identifiable triggers. Depression is common, and anxiety may present with autonomic signs such as tachycardia or sweating. Other notable symptoms include insomnia and unexplained physical complaints such as headaches or pain. Diagnosing IDD is challenging due to its fluctuating and non‐seizure‐related presentation. It must be differentiated from periictal mood changes. Prodromal dysphoric symptoms in the periictal state may precede seizures by hours or days, whereas postictal manifestations, such as depression or anxiety, typically resolve within 24 h.^73^

##### Historical context and definitions

The association between epilepsy and depressive symptoms was first noted by Hippocrates. Kraepelin later described epilepsy‐related dysphoria characterized by irritability, fear, and anxiety. Bleuler expanded the concept to include emotional disturbances and somatic complaints. Some authors have proposed a refined diagnostic framework for IDD, emphasizing mood instability, irritability, and physical symptoms as defining features of IDD.^74^ However, the validity of IDD as a distinct clinical entity and its specificity to epilepsy have been questioned.^75^, ^76^ These debates highlight the need for further empirical clarification regarding the concept of IDD.

##### Treatment

Evidence suggests that antidepressants, including SSRIs and tricyclics, can be effective for treating IDD without increasing seizure frequency, particularly in patients with temporal lobe epilepsy.^77^ ASMs like valproic acid and lamotrigine can stabilize mood while controlling seizures. Accurate diagnosis is essential to avoid misdiagnosing IDD as bipolar disorder or generalized anxiety disorder, which would lead to inappropriate treatment.

## SOCIAL SUPPORT: PSYCHOSOCIAL EDUCATION FOR IMPROVING QOL AND PROMOTING SOCIAL PARTICIPATION

The QOL of PWE is influenced not only by medical management of seizures but also by a complex interplay of psychosocial factors. Comorbid psychiatric symptoms, such as anxiety and depression, along with self‐stigma and social isolation, can profoundly impact overall well‐being.^78^, ^79^ Among these factors, social support plays a pivotal role, with evidence suggesting that positive social interactions considerably enhance QOL in PWE.^80^

### Role and effectiveness of psychosocial education

Psychosocial education aims to provide PWE and families with comprehensive knowledge about epilepsy, management of psychiatric symptoms, stress coping strategies, and development of social skills. It supports reduction of self‐stigma, enhancement of self‐efficacy, and promotion of a positive self‐image.^79^

The Modular Service Package Epilepsy (MOSES), developed in German‐speaking countries, is a structured educational program designed to improve knowledge, coping skills, and self‐management in adults with epilepsy.^81^, ^82^ A randomized controlled trial of MOSES demonstrated significant improvements in knowledge, coping, seizure frequency, and satisfaction with ASM therapy. MOSES comprises nine modules and has been implemented in various healthcare settings. A Japanese adaptation of MOSES, reported by Yamazaki et al. is currently in use in several epilepsy care hospitals and clinics.^83^ An English adaptation, Self‐Management Education for People with Poorly Controlled Epilepsy (SMILE UK), implemented in the United Kingdom, targeted similar goals, although outcomes were mixed.^84^ A personalized epilepsy education intervention for adolescents considerably improved epilepsy knowledge, attitudes, self‐efficacy, and psychosocial outcomes, highlighting the potential of education to address these domains effectively.^85^ Shorter interventions, such as the “one‐day Epi‐school,” have been effective in increasing epilepsy‐related knowledge but may be limited in addressing deeper issues such as self‐stigma and self‐esteem.^79^ Although self‐management education programs have demonstrated benefits in improving knowledge, behaviors, and seizure frequency in adults, the evidence remains variable, limiting generalizability.^86^

Self‐stigma, characterized by internalized negative beliefs such as “I am different from others,” can exacerbate psychiatric symptoms and hinder social interactions.^87^ Psychosocial education assists individuals in confronting these perceptions and building a positive self‐concept. It also equips PWE with practical skills for medication adherence and management of triggers such as stress and sleep deprivation. Family members and caregivers serve as vital sources of emotional and practical support. Psychosocial education empowers them to better understand epilepsy and associated psychiatric symptoms, enhancing their ability to provide effective care. These programs are invaluable for improving knowledge, reducing stigma, and supporting self‐management, although further research is necessary to optimize their psychological and emotional benefits.

### Social welfare support for PWE

Social welfare support is critical to improving QOL and facilitating integration into society. In Japan, PWE can obtain a driver license under specific conditions, such as being seizure‐free for ≥2 years, reflecting efforts to balance independence with safety concerns.^88^, ^89^ Welfare systems provide medical expense subsidies, disability certificates, and pension support to ensure access to treatment and financial stability.^89^ The Services and Support for Persons with Disabilities Act allows for compensation of psychiatric outpatient care and access to disability pensions. In addition, the Mental Health Welfare Notebook broadens eligibility for services, including home care and employment assistance.^90^

Social support is equally critical for epilepsy self‐management, with parents, family members, and significant others often serving as primary supporters. These individuals frequently assist with medication reminders and the implementation of daily routines.^91^ Comprehensive care must extend beyond seizure control to address mental, psychiatric, and physical health, fostering holistic well‐being.^90^ Integration of medical treatment, financial assistance, and robust social support aims to empower PWE to lead fulfilling and independent lives while minimizing the impact of epilepsy on daily activities.

### Future directions in social support for PWE

Future directions in comprehensive epilepsy care emphasize the need for enhanced social support systems to improve QOL and societal participation of PWE. Regional disparities in access to psychosocial services remain a major challenge, as rural areas often lack resources compared to urban centers.^92^, ^93^ Decentralization of resources, expansion of telehealth initiatives, and promotion of mobile health clinics can help address these inequities and ensure equitable access to education, mental healthcare, and vocational rehabilitation.

The persistent stigma surrounding epilepsy continues to hinder social integration and self‐esteem in PWE. Public education campaigns, school‐based awareness programs, and community engagement efforts are essential to normalize epilepsy and dispel misconceptions. Empowerment of PWE through targeted psychosocial education can further mitigate self‐stigma and enhance confidence and social interaction.

Interdisciplinary collaboration among healthcare providers, educators, social workers, and policymakers is critical for effective support. Establishment of standardized communication protocols and creation of integrated care networks can bridge service delivery gaps and promote holistic care. Training professionals to address medical, psychological, and social dimensions of epilepsy management is essential for a unified approach. Therefore, it is important to expand individualized and group‐based psychosocial education programs. These programs should include strategies for managing psychiatric symptoms, improving vocational skills, and fostering community reintegration. Digital platforms can enhance accessibility and engagement, enabling more PWE to benefit from these interventions. Promotion of educational continuity and vocational rehabilitation is crucial for supporting autonomy and fulfillment.^94^, ^95^ Collaboration among schools, healthcare providers, and employers can help PWE overcome barriers to education and employment.^96^ Utilization of welfare systems such as disability pensions and public assistance provides stability and enables broader societal participation.

Advancing psychiatric and psychosocial care in epilepsy requires targeted policy changes and research to develop innovative support models. Long‐term studies are necessary to evaluate existing programs and identify best practices, while advocacy efforts must focus on enhancing the rights and inclusion of PWE. Addressing these challenges will allow epilepsy care to continue evolving, ensuring comprehensive and equitable support for PWE to lead fulfilling lives.

## INDIVIDUALIZED COLLABORATIVE CARE

Thus far, we have examined the diagnosis and treatment of psychiatric symptoms comorbid with epilepsy, as well as support and interventions for associated psychosocial challenges. In this chapter, in order to further clarify the role of psychiatrists, we focus on collaborative practices with individual medical specialties.

### Collaboration with pediatrics

Collaboration between pediatrics and psychiatry is essential for epilepsy care. In particular, psychiatric support for PWE with comorbid developmental disorders, as well as the transition from pediatric to adult healthcare, are critical issues.

#### Psychiatric support for PWE and developmental disorders

Children with epilepsy have a high prevalence of comorbid neurodevelopmental disorders: 17.2% have autism spectrum disorder (ASD), 7.8% have attention‐deficit/hyperactivity disorder (ADHD), and 14.3% have intellectual disability.^97^ These conditions are often associated with difficulties in behavioral regulation and psychiatric symptoms, necessitating collaboration with psychiatry for appropriate diagnosis and support. Notably, the diagnosis of ASD diagnosis is often delayed by an average of 2.5 years from the first parental concern,^98^ highlighting the importance of minimizing the time to formal diagnosis to enable timely intervention. Once diagnosed, individuals may benefit from welfare services, school accommodations, behavioral therapy, social skills training, and parental training.

Pharmacological interventions for neurodevelopmental disorders include antipsychotics and ADHD medications. For children with epilepsy, ADHD medications do not necessarily increase seizure risk. A study comparing hospitalization rates due to seizures between medicated (*n* = 18,166) and non‐medicated individuals (*n* = 54,197) showed no significant difference.^99^ Regarding antipsychotics, one study found that PWE who received these medications had a lower likelihood of seizure exacerbation than those who did not.^43^ Therefore, the potential seizure risk associated with these medications may not be substantial. Nevertheless, close collaboration between pediatrics and psychiatry is essential to ensure appropriate medication adjustments.

#### Transition from pediatric to adult health care

As PWE reach adulthood, they must transition from pediatric to adult care, including neurology, psychiatry, and neurosurgery. However, several factors complicate this process.

From the perspective of patients and their families, those who have been under the long‐term care of a pediatrician may feel apprehensive about transitioning to a new medical team. One study found that 73% of parents of children with epilepsy expressed concerns about the adequacy of care transition, and 59% preferred continued pediatric care.^100^ In addition, patients with developmental disorders often face difficulties adapting to new environments,^101^ further complicating the transition.

Differences in clinical culture also contribute to the challenges. Pediatricians often serve as generalists, addressing a broad range of needs in children.^102^ In contrast, adult care may require patients to visit multiple specialized departments for similar services. Moreover, while pediatric care emphasizes parental involvement and the perspective of the child,^103^ adult psychiatric care prioritizes building rapport directly with the patient.^104^ Another issue is that adult care providers may be less familiar with pediatric epilepsy syndromes.^105^ Consequently, >30% of patients aged >15 years continue to receive care in pediatric settings.^106^

To address these challenges, it is essential to educate patients and families early about the need for transition and implement a structured plan. A systematic approach involving gradual psychoeducation beginning around age 12 and a progressive shift in treatment responsibility from the family to the patient has been established to facilitate a smooth transition.^107^

### Collaboration with neurosurgery

Collaboration between neurosurgeons and psychiatrists is essential for providing comprehensive care in epilepsy management. Surgical intervention plays a pivotal role for patients with drug‐resistant epilepsy. In such cases, surgery often offers the most effective means of achieving seizure control and considerably enhances the patient's QOL.^108^ However, psychiatric issues frequently emerge during the surgical process, necessitating interdisciplinary collaboration to address both neurological and psychiatric factors. Key areas of focus include managing preoperative psychiatric symptoms, conducting thorough preoperative evaluations, and addressing postoperative psychiatric complications. These efforts are critical for optimizing outcomes and ensuring holistic patient care.

#### Preoperative psychiatric symptoms

Anxiety is a common preoperative issue due to the invasive nature of epilepsy surgeries. Procedures such as intracranial electrode placement, often used for diagnostic purposes, can exacerbate psychological distress. Patients may also experience pain during these tests, and the reduction or discontinuation of ASM for seizure monitoring can further increase anxiety. Enforced bed rest to prevent seizure‐related injuries adds to the emotional burden. Collectively, these factors contribute to considerable preoperative anxiety and insomnia, requiring timely management.

The evaluation of seizure‐related psychotic symptoms is also crucial. Psychotic symptoms directly linked to seizures often indicate the suitability for surgical intervention.^109^ However, neurosurgeons may hesitate to proceed due to limited psychiatric expertise. In such cases, psychiatrists should advocate for surgery when appropriate and provide clear assessments to guide decision‐making. Conversely, psychiatrists should not hesitate to evaluate patients presenting with seizure‐related psychotic symptoms to ensure accurate diagnosis and care.

#### Postoperative psychiatric symptoms

Postoperative psychiatric complications include acute psychosis, mood disturbances, and suicidal ideation. Acute psychosis may arise following resection surgery, including the rare condition of post‐lobectomy epilepsy psychosis, which has a reported prevalence of approximately 1.1%.^110^ Psychosis typically occurs within a year of surgery in patients with persistent seizures or complications and may manifest as persecutory delusions, auditory hallucinations, and residual seizures. Risk factors include bilateral cerebral abnormalities and a smaller amygdala on the non‐resected side. Psychosis in patients without a prior psychiatric history is referred to as de novo psychosis. These conditions require immediate intervention.

Mood disturbances are common and may range from major depressive episodes to subtle symptoms, which may be misinterpreted as personality traits or environmental reactions. Suicidal ideation can also occur in patients with mild depressive symptoms. Suicide after surgery is recognized as a serious risk,^111^ and requires close, continuous monitoring. A thorough preoperative evaluation is essential for differentiating postoperative psychiatric symptoms from preexisting conditions. Identifying personality traits, neurodevelopmental factors, and psychiatric history enables targeted care and reduces the likelihood of overlooking major postoperative changes.

#### Tips for effective collaboration with neurosurgeons

To address this challenge, psychiatrists must adopt a proactive role in perioperative care, provide timely consultations, and actively contribute to policy discussions. By ensuring that psychiatric considerations are integrated into treatment plans, psychiatrists can help improve outcomes and enhance the quality of care for PWE.

### Collaboration with neurology

#### Diagnostic challenges and the position of PNES between disciplines

Collaboration between neurology and psychiatry in epilepsy care includes the treatment of psychiatric symptoms in PWE and the differentiation between ES and psychiatric disorders. This section focuses on PNES.

PNES is a term originating from the field of epilepsy and is classified as a dissociative neurological symptom disorder in the ICD‐11, a dissociative disorder or conversion disorder (functional neurological symptom disorder) in the Diagnostic and Statistical Manual of Mental Disorders‐5, and a subtype of functional neurological disorder in neurology.^112^ PNES is also important as a differential diagnosis for epilepsy, though it frequently co‐occurs with epilepsy: 22% of PNES cases also have epilepsy, and 12% of epilepsy cases also present with PNES.^113^ Therefore, PNES lies at the intersection of psychiatry and neurology, and collaboration between these specialties is highly desirable. However, in practice, PNES is often not well accepted by either department, and responsibility for treatment is frequently disputed.^114^

Psychiatry typically diagnoses PNES based on life history and personality traits, such as trauma and stress, which may contribute to symptom development,^112^ whereas neurologists approach diagnosis differently. Neurological diagnosis relies on seizure semiology and electrophysiological data. Certain clinical signs, such as eye closure during seizures, prolonged duration, and clusters of seizures with intervening cessation,^115^ can aid in differential diagnosis even for non‐epileptologists. However, diagnosis should not be based on a single symptom.

Although the presence or absence of interictal epileptic discharges is not useful in diagnosing PNES and carries a risk of misdiagnosis, the gold standard is Long‐Term Video EEG monitoring (LTVEM) to record and analyze seizure episodes.^116^ However, in some cases, seizure events are not captured even with LTVEM, or access to such monitoring may be limited. Consequently, diagnosis is often made based on clinical interviews alone.

#### Psychiatric involvement and the importance of acceptance and engagement

When a psychiatrist is consulted by a neurologist regarding a patient with suspected PNES, it is crucial to determine the diagnostic certainty level, as defined by LaFrance et al.^115^ The diagnostic framework proposed by LaFrance et al. includes four levels of certainty—Possible, Probable, Clinically Established, and Documented—based on combinations of clinical history, witnessed events, and EEG findings, with ‘Documented’ representing the highest level of diagnostic certainty and ‘Possible’ the lowest ^115^.

A “Documented” diagnosis refers to a typical event captured during LTVEM, which a clinician experienced in seizure disorders identifies as PNES in nature. In contrast, a ‘Possible’ diagnosis is made when no clinician has directly observed the event, but the clinical history strongly suggests PNES and interictal EEG shows no epileptiform activity.

The purpose of determining the diagnostic level is not to question the validity of the diagnosis per se, but to understand how the patient's and family's acceptance and comprehension of the diagnosis may relate to its level of certainty—an important consideration with prognostic implications.

Knowing whether the diagnosis is Possible, Probable, Clinically Established, or Documented can significantly inform the psychiatric assessment and care planning. In particular, even in cases of Documented PNES, patients may struggle to accept the diagnosis. In such situations, psychiatrists should provide targeted psychoeducation to support diagnostic acceptance and engagement in appropriate treatment.

Conversely, in cases of Possible PNES, the diagnosis remains provisional and may ultimately prove to be incorrect. Psychiatrists should therefore adopt a cautious and exploratory approach, avoid premature diagnostic closure, and collaborate closely with neurologists to ensure an accurate differential diagnosis.

Although individualized support and psychotherapy are essential for treating PNES, acceptance of the diagnosis is a critical first step.^116^ In some cases, PNES resolves after a diagnostic explanation alone, without any special intervention.^117^

Regardless of diagnostic certainty, psychiatrists should continue consultation. Some psychiatrists may refer patients back to neurologists after one visit if no obvious psychogenic factors are identified. However, PNES psychogenesis is often complex and may only become evident over time. Many patients report distress from being ping‐ponged between specialists.^118^ Therefore, simply maintaining a willingness to engage in ongoing care can itself have therapeutic value.

#### Psychotherapeutic strategies and interdisciplinary follow‐up

Patients should be encouraged to record daily life and seizure episodes using a life chart, which is employed for conditions like depression and sleep disorders. Stress coping skills should also be taught while reviewing the pattern and triggers of PNES based on the life record at each visit.

A systematic review and meta‐analysis of cognitive behavioral therapy (CBT) for PNES showed that CBT is significantly associated with seizure freedom, reduced anxiety, and improved QOL.^119^ If a psychiatrist is trained in CBT, it should be used to help patients recognize and reframe thought patterns, manage stress, and prevent seizures.

Building a trusting therapeutic relationship is the next step. Once complex contributing factors and those maintaining PNES are identified, environmental adjustments should be explored through a multidisciplinary approach.

As PNES symptoms may fluctuate, sometimes worsening temporarily during therapy, it is advisable to maintain collaborative follow‐up with the neurologist. Ideally, this should continue until antiseizure medications are tapered or for at least one year.

In summary, PNES treatment requires both a neurologist's diagnostic expertise and a psychiatrist's therapeutic perspective. Ideally, both departments should integrate these roles within their respective domains.

## FUTURE PERSPECTIVES

As discussed in the Introduction, the epilepsy care system in Japan is at a turning point. Historically, psychiatrists have played a central role, and Japan has made important contributions to the understanding of epileptic psychosis. However, recent decades have seen a decline in psychiatric involvement, with insufficient attention paid to broader psychosocial issues such as depression, anxiety, and functional seizures. Although global standards in epilepsy care increasingly emphasize QOL and psychiatric comorbidities, Japan is at risk of falling behind unless systemic changes are implemented. In response, a renewed clinical framework is introduced from a psychiatric perspective and proposed as a practical approach to redefine and revitalize the role of the psychiatrist in epilepsy care.^13^

Several urgent challenges must be addressed to realize this vision. First, there is a serious gap between policy and clinical reality. Although epilepsy is still categorized as a psychiatric disorder in the administrative system of Japan, most psychiatrists feel unprepared to manage it. A national survey showed that approximately 90% of psychiatrists reported discomfort in treating epilepsy because of limited training and exposure.^5^ This has led to the underdiagnosis and undertreatment of psychiatric symptoms among PWE, especially in areas with limited access to epilepsy specialists. Several strategies must be considered to address these issues.

### Educational transformation

Psychiatric training should be updated to include epilepsy neurology, seizure classification, and the bidirectional relationship between epilepsy and mental health. Continuing medical education should consist of joint case discussions with neurologists and clinical exposure to conditions such as IF and PIP. Creating certification programs in the psychiatric aspects of epilepsy care may help psychiatrists reengage with epilepsy care in a defined and supported manner.^120^

### Clinical integration and redefining roles

This renewed framework does not require psychiatrists to manage seizures directly. Rather, they should focus on diagnosing and treating comorbid psychiatric conditions and providing psychosocial support. Multidisciplinary teams involving neurologists, neurosurgeons, and psychiatrists should be promoted through models such as co‐managed PNES clinics and embedded psychiatric care in epilepsy centers. Japan's historical model, the 1975 Comprehensive Epilepsy Center, offers a blueprint for such collaborative systems. This vision is in alignment with recent initiatives of the ILAE Psychiatry Commission, which has emphasized the development of integrated mental health care pathways tailored for PWE.^9^ These efforts advocate for the embedding of psychiatric assessment and intervention within routine epilepsy care, promoting not only collaboration between neurologists and psychiatrists but also involvement of clinical psychologists, nurses, and social workers. Such multidisciplinary approaches are essential to address the full spectrum of psychiatric, cognitive, and social needs of PWE and to ensure continuity of care across the lifespan.

### Advancing research and policy reform

Japan's contributions to epilepsy psychosis research are notable; however, attention to other relevant areas, such as depression, PNES, and stigma, remains limited. To address these issues, national funding bodies should support large‐scale studies and clinical guidelines. Policy reforms are also required to reclassify epilepsy within the healthcare system as a neurological disorder with psychiatric comorbidities, thereby enabling better coordination between specialties and reducing administrative inconsistencies.

### Expanding community‐based care and public engagement

Stigma remains a major barrier to care worldwide.^121^ Psychiatrists should spearhead public education campaigns that redefine epilepsy not as a purely psychiatric illness but as a complex neurological condition with psychosocial dimensions. School‐based programs, employment support initiatives, and family‐centered interventions must be developed and guided by psychiatric experts. Just as survivorship campaigns have reshaped perceptions of cancer, epilepsy must be humanized and destigmatized through patient narratives and media collaboration.

### Strengthening global ties and institutional sustainability

To avoid professional isolation, Japanese psychiatrists should participate in global dialogues, including with the ILAE Neuropsychiatry Commission. Universities and academic centers should establish dedicated programs for the psychiatric and psychosocial dimensions of epilepsy, including research hubs and clinical fellowships. Senior psychiatrists must actively mentor younger generations to ensure the continuity of expertise over time.

The solution lies not in lamenting the marginalization of psychiatry in Japan's epilepsy care, but in revisiting the historical foundations of neuropsychiatry and re‐establishing the psychiatrist's essential role in shaping the future of comprehensive epilepsy treatment. treatment. By prioritizing QOL, addressing psychiatric comorbidities, and promoting interdisciplinary collaboration, Japan has the potential to reclaim a leadership position in epilepsy research and clinical practice. Achieving this goal will require more than just technical reforms—it will demand a fundamental cultural shift in how epilepsy is understood and treated. Now is the time for concerted action—through educational reform, policy development, clinical integration, and public engagement—to establish a future in which psychiatrists assume a central role in the comprehensive care of PWE.

## AUTHOR CONTRIBUTIONS

**Go Taniguchi**: conceptualization; data curation; project administration; writing — review and editing. **Hirotaka Iwaki**: writing — review and editing. **Izumi Kuramochi:** writing — review and editing**. Toru Horinouchi**: writing — review and editing**. Shunsuke Takagi**: writing — review and editing.

## CONFLICT OF INTEREST STATEMENT

The authors declare no conflicts of interest.

## ETHICS APPROVAL STATEMENT

This review article does not contain any studies with human participants, and therefore, ethical approval was not required.

## PATIENT CONSENT STATEMENT

This review article does not involve any individual patient data, and therefore, patient consent was not required.

## CLINICAL TRIAL REGISTRATION

N/A.

## Data Availability

Raw data were generated at the Department of Epileptology, National Center of Neurology and Psychiatry. Derived data supporting the findings of this study are available from the corresponding author G. T. on request.
